# Expression of Tim-3 on neutrophils as a novel indicator to assess disease activity and severity in ankylosing spondylitis

**DOI:** 10.3389/fmed.2025.1530077

**Published:** 2025-03-13

**Authors:** Xuechan Huang, Yuebing He, Guanqun Yi, Shaoling Zheng, Weiming Deng, Shuyang Chen, Ruiqi Zhu, Yunqing Wang, Junming Chen, Chun Zheng, Zhixiang Huang, Tianwang Li

**Affiliations:** ^1^Department of Rheumatology and Immunology, The Affiliated Guangdong Second Provincial General Hospital of Jinan University, Guangzhou, China; ^2^The Second School of Clinical Medicine, Southern Medical University, Guangzhou, China; ^3^Department of Rheumatology and Immunology, Zhaoqing Central People's Hospital, Zhaoqing, China

**Keywords:** ankylosing spondylitis, neutrophils, Tim-3, disease activity, immunomodulation

## Abstract

**Objective:**

To investigate the expression of Tim-3 on neutrophils in ankylosing spondylitis (AS) patients and its correlation with disease activity, severity, and inflammatory markers.

**Methods:**

Sixty-two AS patients from Guangdong Second Provincial General Hospital and 38 healthy controls (HC) were enrolled. Clinical data, physical exams, and laboratory measurements were recorded. Flow cytometry measured Tim-3 and PD-1 expression on neutrophils, real-time PCR quantified mRNA levels and protein expression of Tim-3 was determined by Western blot. We analyzed the correlation between Tim-3 mean fluorescence intensity (MFI) on neutrophils, inflammatory markers, and AS disease activity and severity.

**Results:**

Tim-3 expression on neutrophils was higher in AS patients than in HC, showing a positive correlation with erythrocyte sedimentation rate (ESR), c-reactive protein (CRP), and Ankylosing Spondylitis Disease Activity Score (ASDAS). Active AS patients (ASDAS ≥ 1.3) had increased Tim-3 MFI compared to inactive ones (ASDAS < 1.3). Regular treatment with non-steroidal anti-inflammatory drugs (NSAIDs), biological disease-modifying anti-rheumatic drugs (bDMARDs), and conventional synthetic disease-modifying anti-rheumatic drugs (csDMARDs) over a month significantly reduced Tim-3 MFI in AS patients.

**Conclusion:**

Elevated Tim-3 expression on neutrophils correlates with increased inflammatory markers and AS activity. Treatment lowered Tim-3 MFI, suggesting its potential as an indicator for assessing AS disease activity and severity and as a feedback mechanism to reduce tissue damage from inflammation.

## 1 Introduction

Ankylosing spondylitis (AS) is a chronic, prevalent inflammatory rheumatic disease affecting the axial skeleton and sacroiliac (SI) joints, characterized by inflammatory low back pain, progressive spinal stiffness, and the potential for spinal immobility and ankylosis, which can significantly impair quality of life over time. Additionally, the involvement of peripheral joints, digits, and entheses are hallmark features of this disease (1). The etiology of AS involves a complex interplay between genetic predisposition and environmental factors. Approximately ninety to ninety-five percent of AS patients test positive for HLA-B27 (human leukocyte antigen B27), underlining a significant familial hereditary tendency. Furthermore, factors such as the gut microbiome, sex hormones, and Vitamin D levels have been implicated in the development of AS (2, 3). Despite advances in medical knowledge, AS remains a formidable challenge in clinical practice, and its pathogenic mechanisms have yet to be fully elucidated (4). Both innate and adaptive immunity are recognized to play pivotal roles in the development and progression of AS. Previous studies have highlighted the involvement of T cells, B cells, macrophages, and natural killer (NK) cells in the pathogenesis of AS (5–7).

In recent decades, emerging evidence has unequivocally underscored the pivotal roles of neutrophils in the progression of AS. Studies have reported increased in neutrophil counts in AS patients, particularly during active periods (8, 9). Moreover, alterations in the functions of neutrophils, including migration, phagocytosis, and respiratory burst activity, have been observed in AS (10, 11). Specifically, AS patients have exhibited heightened neutrophil migration compared to HC (12). The respiratory burst activity of neutrophils has been implicated in tissue and cartilage damage, potentially contributing to the pathophysiology of AS (13, 14). Nonetheless, the precise role of neutrophils in AS pathogenesis remains incompletely elucidated.

T cell immunoglobulin and mucin-domain containing molecule 3 (Tim-3), an immune checkpoint molecule, is constitutively expressed on T cells, macrophages/monocytes, natural killer cells, and dendritic cells (15). Recent findings have revealed that Tim-3 is also present on neutrophils and can modulate the inflammatory response via the ligand galectin-9 (Gal-9) in cystic fibrosis lung disease (15). Previous data suggest that Tim-3 negatively regulates the activation and/or function of immune cells, potentially influencing the progression of autoimmune diseases. Some research indicates that Tim-3 may serve as a marker for dysfunctional macrophages, monocytes, dendritic cells, CD4+, and CD8+ T cells (15). A decrease in Tim-3+ Treg in AS has been directly correlated with the Bath ankylosing spondylitis disease activity index (BASDAI) score, CRP level, and ESR (16). Previous research suggests that Tim-3 regulates the function of various T cell subsets, making it a potential new marker for assessing the severity of rheumatoid arthritis (RA) (17). However, the frequency and roles of Tim-3 expression on neutrophils in AS have not been established. In our study, we examined the expression of Tim-3 on neutrophils in AS patients and analyzed whether Tim-3-expressing neutrophils correlated with the activity and severity of AS.

## 2 Materials and methods

### 2.1 Ethical statement

This study was approved by the Ethics Committee of Guangdong Second Provincial General Hospital, Guangdong, China (2018-FSKWZ-010, 2021-KZ-195-01). Written informed consent about the experimental requirements and potential risks was provided by all participants before they entered the study.

### 2.2 Subjects

In this study, we recruited 62 AS patients who met the modified New York criteria (18). They were enrolled in the Guangdong Second Provincial General Hospital from January 2019 to July 2020. Those who had malignancy, active infection, diabetes mellitus, hypertension, renal failure, and liver failure were excluded from the study. In addition, 38 healthy volunteers without inflammatory or autoimmune diseases were included in this study. The characteristics of the study subjects are presented in Table 1.

**Table 1:** Baseline characteristics of ankylosing spondylitis.

	**AS patients (*n* = 62)**	**Healthy controls (*n* = 38)**
Male/female, male %	47/15(76%)	22/16(58%)
Age, years, average ± SEM	34 ± 11	37 ± 14
Leucopenia	0	
Erythrocytopenia	14	
Thrombocytopenia	0	
Anemia	17	
Elevated ESR	35	
Elevated CRP	33	
ASDAS, median	2.99	

Baseline characteristics of patients with ankylosing spondylitis (AS) and healthy controls. Data are presented as counts or mean ± SEM. Male % represents the proportion of male participants in each group. Leucopenia, Thrombocytopenia, Erythrocytopenia and anemia indicate the number of patients diagnosed with these conditions. Elevated ESR and CRP indicate the number of AS patients with increased erythrocyte sedimentation rate and C-reactive protein levels, respectively. ASDAS represents the Ankylosing Spondylitis Disease Activity Score, with median values reported.

### 2.3 Clinical and laboratory assessments

Gender, age, clinical features, HLA-B27 values, number of white blood cells (WBCs), neutrophils, lymphocytes, monocytes, red blood cells (RBCs), hemoglobin level, platelets, CRP level, and ESR level were recorded.

Disease activity was assessed by the BASDAI, Bath Ankylosing Spondylitis Functional Index (BASFI), and ASDAS (19).

### 2.4 Isolation of human neutrophils

Neutrophils were obtained from peripheral whole blood (about 5 ml) of HC and AS patients by Ficoll density gradient centrifugation as described previously (20, 21). Cell preparations yielded > 95% neutrophils.

### 2.5 Flow cytometry analysis

In the flow cytometry analysis, we analyzed the molecular phenotype of neutrophils. Antibodies used included FITC labeled anti human CD66b (Ebioscience, San Diego, USA), PE/CY7 labeled anti human CD366 (Tim-3) (Biolegend, San Diego, USA), and APC/CY7 labeled anti human CD270 (PD-1) (Biolegend, San Diego, USA). Isotype control was set up in the experiment, and cells were incubated with PE and FITC labeled mouse IgG isotype antibodies to evaluate nonspecific binding. To eliminate the spectral overlap in multicolor fluorescence detection, we used single staining controls (FITC, PE/CY7, and APC/CY7) to adjust fluorescence compensation. In addition, we also used fluorescence minus one control (FMO) to determine the gating boundary of Tim-3 and PD-1 expression. The FMO controls contains all other fluorescent markers except the target fluorescent dye. Data were analyzed using FCS express 7 and flowjo 10.7, and statistics and chart generation were done using graphpad prism 9.

### 2.6 Real-time quantitative polymerase chain reaction

The expression levels of Tim-3 and PD-1 to β-actin RNA were measured with the LightCycler^®^ system (Roche, Mannheim, Germany). The total RNA was isolated from neutrophils with trizol extraction. According to the manufacturer's instructions, the first-strand cDNA synthesis kit (TOYOBO, Japan) for real-time polymerase chain reaction (RT-PCR) was used to prepare the first-strand cDNA. Primers were designed by Primer Premier 5.0 and synthesized by Shanghai Generay Biotech Co., Ltd (Shanghai, China). Primers targeting Tim-3 and PD-1 were as follows: Tim-3 (forward, 5′-TCCAAGGATGCTTACCACCAG-3′; reverse, 5′-GCCAATGTGGATATTTGTGTTAGATT-3′); PD-1 (forward, 5′-CCAGGATGGTTCTTAGACTCCC-3′; reverse, 5′-TTTAGCACGAAGCTCTCCGAT-3′). The β-actin fragment was amplified using the following primer: forward, 5′-TGTTCCCCTTGGTATTTG-3′; reverse, 5′-CAAGACAAAACAACTGGT-3′. The data analysis was performed utilizing LightCycler Software, version 3.5 (Roche), and LinRegPCR program, version 7.5. To adjust for variations in the amount of input RNA, the Tim-3 and PD-1 levels were normalized against the mRNA levels of the β-actin using the calculation 2^−ΔCt^.

### 2.7 Western blot

Neutrophils were isolated from peripheral blood samples of healthy volunteer and AS patient using Ficoll density gradient centrifugation as described in Section 2.4. Total cellular proteins were extracted using RIPA lysis buffer (Beyotime, China) supplemented with protease inhibitors (Roche, Switzerland). Protein concentrations were quantified using a BCA Protein Assay Kit (Thermo Fisher Scientific, USA) according to the manufacturer's instructions. Equal amounts of protein (15 μg per lane) were separated by 10% SDS-PAGE and transferred onto polyvinylidene fluoride (PVDF) membranes (Millipore, USA). The membranes were blocked with blocked buffer (Beyotime, China) in Tris-buffered saline containing 0.1% Tween-20 (TBST) for 1 h at room temperature and then incubated overnight at 4°C with a primary antibody against TIM-3 (anti-TIM-3, Proteintech, USA; dilution 1:1,000). After washing with TBST, the membranes were incubated with a horseradish peroxidase (HRP)-conjugated secondary antibody (Cell Signaling Technology, USA; dilution 1:2,000) for 1 h at room temperature. Protein bands were visualized using an enhanced chemiluminescence (ECL) detection system (Bio-Rad, USA). GAPDH (anti-GAPDH, Cell Signaling Technology, USA; dilution 1:1,000) was used as an internal control to ensure equal protein loading.

### 2.8 Magnetic resonance imaging scoring

MRI can be used to detect the soft tissue inflammation and active inflammation of hip and sacroiliac joints (SJ) involvement earlier than X-rays in AS patients. The MRI scoring system of the sacroiliac joint or the hip has been applied in AS. The inflammation of the SJ in MRI was assessed by the Spondyloarthritis Research Consortium of Canada (SPARCC) MRI index (22). In short, the MRI assessments of sacroiliitis were performed in six consecutive coronal slices, according to the presence, depth, and intensity of bone marrow edema. The inflammation of the hip in MRI was assessed using the hip inflammation MRI scoring system (HIMRISS) (23). The HIMRISS value for one hip is composed of the values of bone marrow lesion (BML) and synovitis, ranging from 0 to 130. After each side was evaluated, the total scores of the two hips were summed up as the patient's final HIMRISS score.

The radiologist who was blinded to the clinical and laboratory findings analyzed all MRI tests and provided the scores, which were used for the association analysis.

### 2.9 Statistical analysis

SPSS 13.0 was used for statistical analysis, and graphic presentation was performed with GraphPad Prism version 5.0. The *t*-test was employed upon confirmation of normal data distribution; otherwise, the nonparametric Mann–Whitney test was utilized for data analysis. For evaluating changes with treatment in the group of eight patients, the paired *t*-test was conducted. Similarly, either the Pearson method or the nonparametric Spearman method was applied for correlation analysis. Furthermore, sensitivity and specificity were analyzed by the receiver operating characteristic (ROC) curve. Statistical significance was considered to exist when *P* < 0.05.

## 3 Results

### 3.1 Characteristics of study subjects

The characteristics of AS patients and HC enrolled in this study are summarized in Table 1. There were no significant differences observed between patients and HC in terms of age or gender distribution.

### 3.2 Elevated expression of Tim-3 on neutrophils in AS patients

**Figure 1:**
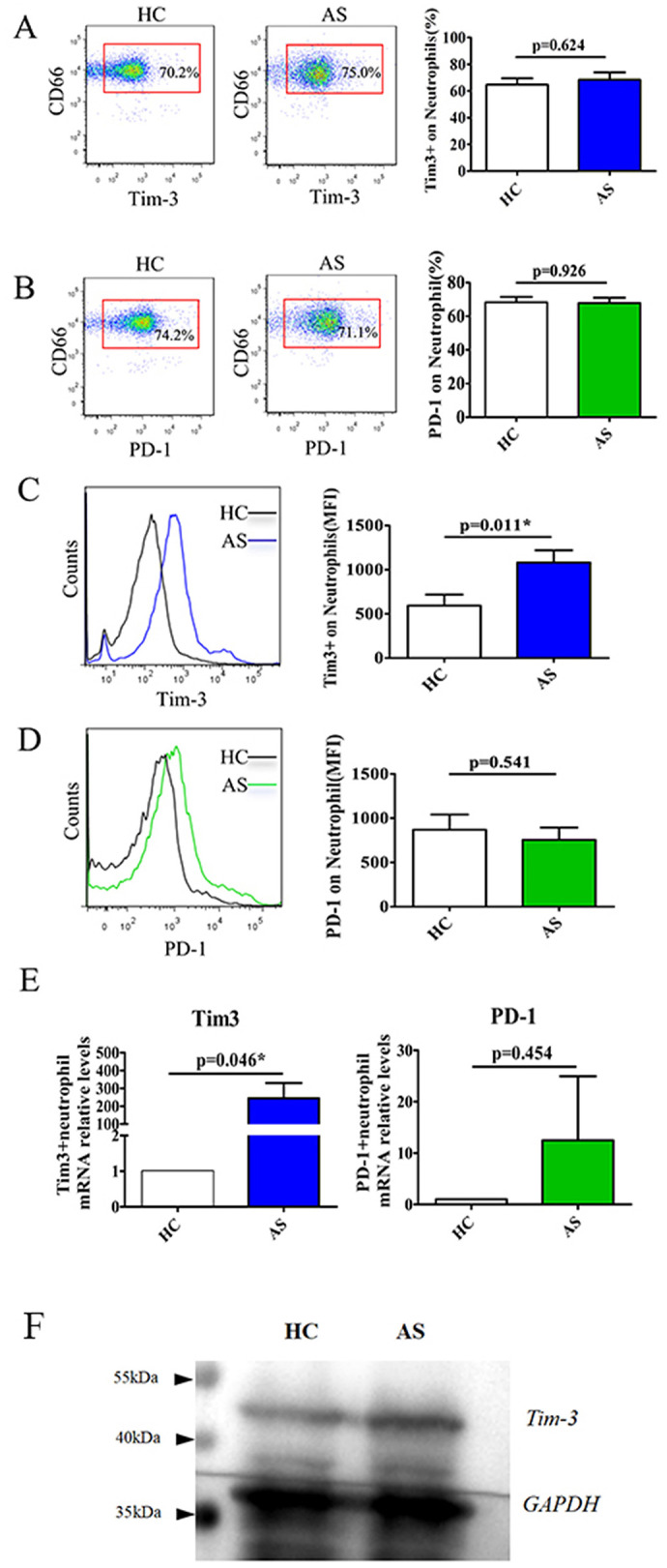
Elevated the expression of Tim-3 on neutrophils in patients with AS. Flow cytometric analysis was used to detect Tim-3 and PD-1 expression in neutrophils **(A–D)**. **(A)** Shows the representative scatter plots of Tim-3 expression on neutrophils (left), horizontal bars indicated the percentage of Tim-3-expressing neutrophils in patients with AS and HC (right). **(B)** Shows the representative scatter plots of PD-1 expression on neutrophils (left), horizontal bars indicated the percentage of PD-1-expressing neutrophils in patients with AS and HC (right). **(C)** The representative plots and the mean fluorescence intensity (MFI) of Tim-3 expression on neutrophils in patients with AS and HC. **(D)** The representative plots and MFI of PD-1 on neutrophils in patients with AS and HC. **(E)** Relative mRNA expression and fold increase of Tim-3 and PD-1 in patients with AS and HC were analyzed by quantitative RT-PCR. *P < 0.05 as compared with HC. **(F)** Western blot analysis of Tim-3 protein expression in neutrophils from healthy volunteer and AS patient.

Neutrophils were identified in peripheral blood as CD66+ populations and analyzed using flow cytometry to assess the expression of costimulatory molecules, including Tim-3 and PD-1. The data revealed that the percentage of Tim-3-expressing neutrophils did not exhibit a significant difference between the AS patients and the healthy group (Figure 1A, *P* = 0.624). However, the MFI of Tim-3 on neutrophils was notably increased in AS patients compared to HC (Figure 1C, *P* = 0.011). Conversely, no significant difference was observed in the frequency of PD-1-expressing neutrophils or PD-1 MFI on neutrophils between AS patients and HC (Figures 1B, D, *P* = 0.926 and 0.541). Furthermore, Tim-3 mRNA levels were found to be significantly elevated in AS patients compared to HC, whereas there was no significant difference observed in PD-1 mRNA levels between AS patients and HC (Figure 1E, *P* = 0.046 and 0.454). Simultaneously, we further verified the protein expression of Tim-3 using Western blot. The results indicate that the total protein expression of Tim-3 in neutrophils from the AS patient is elevated compared to that of the healthy control, which is consistent with the increasing trend observed at the mRNA level (Figure 1F).

### 3.3 The MFI of Tim-3 on neutrophils correlated with markers of inflammation

**Figure 2:**
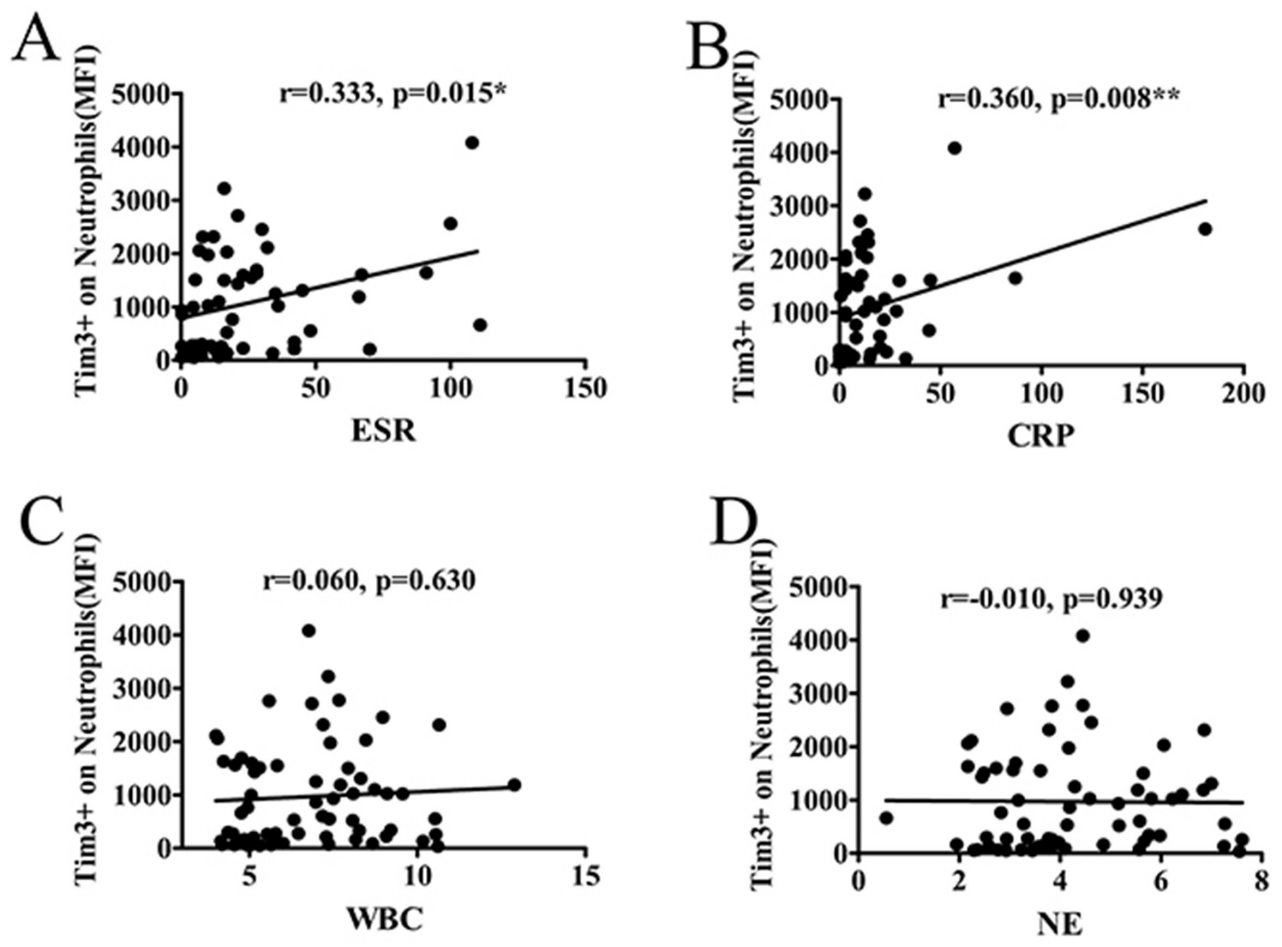
The MFI of Tim-3 on neutrophils correlated with markers of inflammation. **(A)** The MFI of Tim-3 of neutrophils in AS patients correlated significantly with ESR (*r* = 0.333, *P* = 0.015). **(B)** The MFI of Tim-3 of neutrophils in AS patients correlated significantly with CRP (*r* = 0.360, *P* = 0.008). **(C)** The MFI of Tim-3 of neutrophils in AS patients was not associated with WBC (*r* = 0.06, *P* = 0.630). **(D)** The MFI of Tim-3 of neutrophils in AS patients was not associated with neutrophil count (*r* = −0.010, *P* = 0.939). ^*^P < 0.05, ^**^P < 0.01.

AS patients often exhibit elevated levels of inflammatory markers. To explore the association between the level of Tim-3 MFI of neutrophils and inflammatory markers, including ESR, CRP, WBC, and neutrophil count (NE), we conducted analyses in AS patients. The results revealed a positive correlation between the Tim-3 MFI of neutrophils and ESR or CRP (Figures 2A, B). However, the MFI of Tim-3 on neutrophils in AS patients did not exhibit a significant association with WBC or NE (Figures 2C, D). Furthermore, we extended our analysis to other routine blood parameters, including red blood cell (RBC) count, hemoglobin (HGB) levels, hematocrit (HCT), lymphocyte (LY) count, monocyte (MONO) count, and platelet (PLT) count. The findings suggest that Tim-3 expression on neutrophils is not broadly associated with general hematological indices but may be more specifically linked to inflammatory markers such as ESR and CRP in AS patients (Supplementary Figure 1).

### 3.4 The level of Tim-3 MFI of neutrophils was associated with disease activity and severity of AS

The aforementioned findings revealed a correlation between the MFI of Tim-3 on neutrophils and markers of inflammation. Notably, ESR and CRP are conventionally utilized for monitoring disease activity in AS patients. Therefore, AS patients were stratified into two groups: those with active disease (ASDAS ≥ 1.3) and those with inactive disease (ASDAS < 1.3), based on the ASDAS criteria. Subsequently, the relationship between the MFI of Tim-3 on neutrophils and disease activity was analyzed.

**Figure 3:**
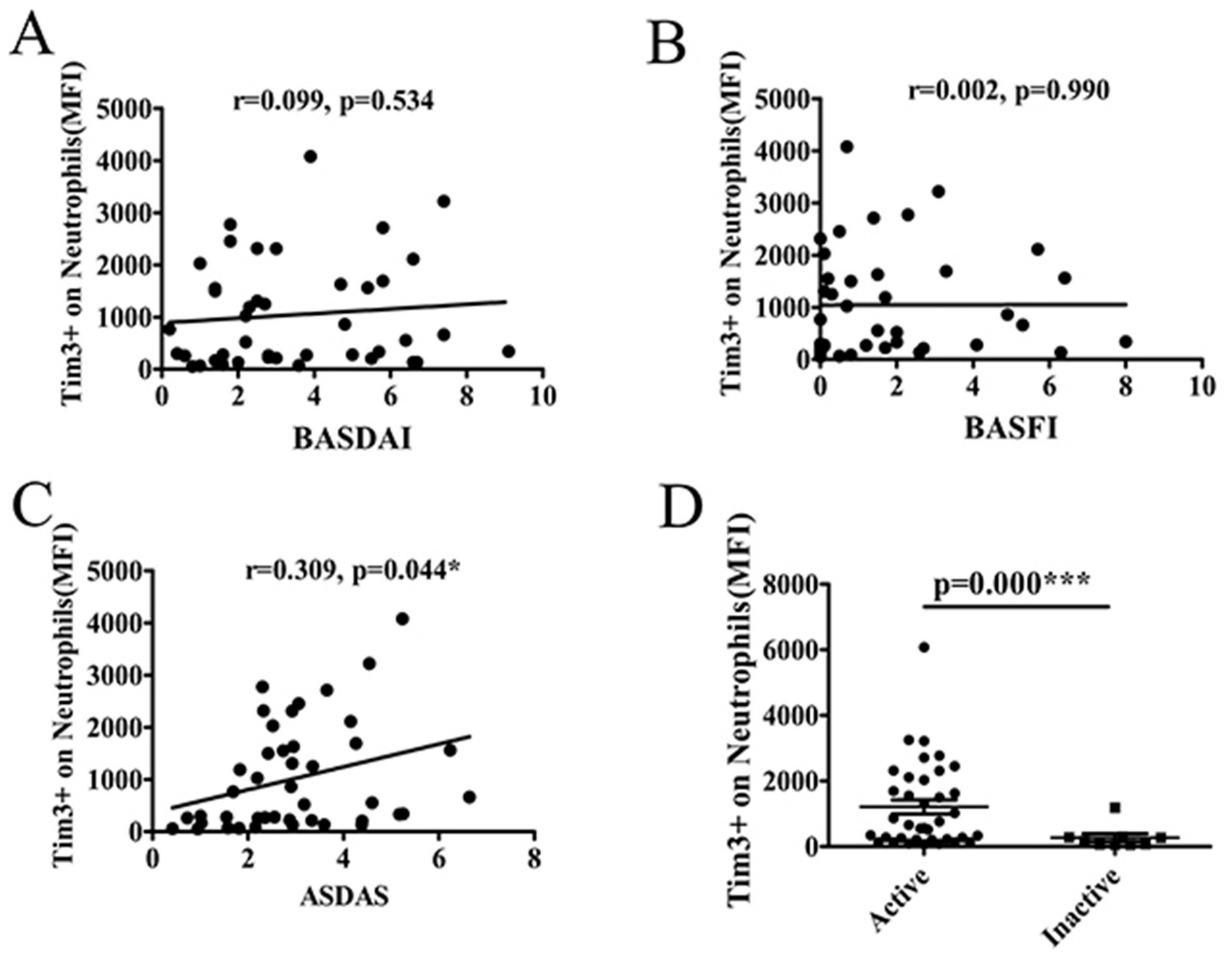
The level of Tim-3 MFI on neutrophils was associated with disease activity and severity of AS. **(A)** The MFI of Tim-3 on neutrophils in AS patients was not associated with BASDAI (*r* = 0.099, *P* = 0.534). **(B)** The MFI of Tim-3 on neutrophils in AS patients was not associated with BASFI (*r* = 0.002, *P* = 0.990). **(C)** The MFI of Tim-3 on neutrophils in AS patients correlated significantly with ASDAS(*r* = 0.309, *P* = 0.044). **(D)** The MFI of Tim-3 on neutrophils in AS patients was significantly increased in active patients compared to those with inactive (*P* =0.000). ^*^P < 0.05, ^***^P < 0.001.

Our analysis revealed a positive correlation between the Tim-3 MFI of neutrophils and the ASDAS score, indicating a link between Tim-3 expression on neutrophils and disease activity in AS. However, no significant correlation was observed between the Tim-3 MFI of neutrophils and BASDAI or BASFI scores (Figures 3A–C). This demonstrated that the Tim-3 MFI of neutrophils was correlated with disease activity in AS. Additionally, the level of Tim-3 MFI on neutrophils was significantly higher in patients with active AS compared to those with inactive AS (Figure 3D).

### 3.5 Tim-3 on neutrophils had a high evaluation value for the severity of AS by ROC curve analysis

**Figure 4:**
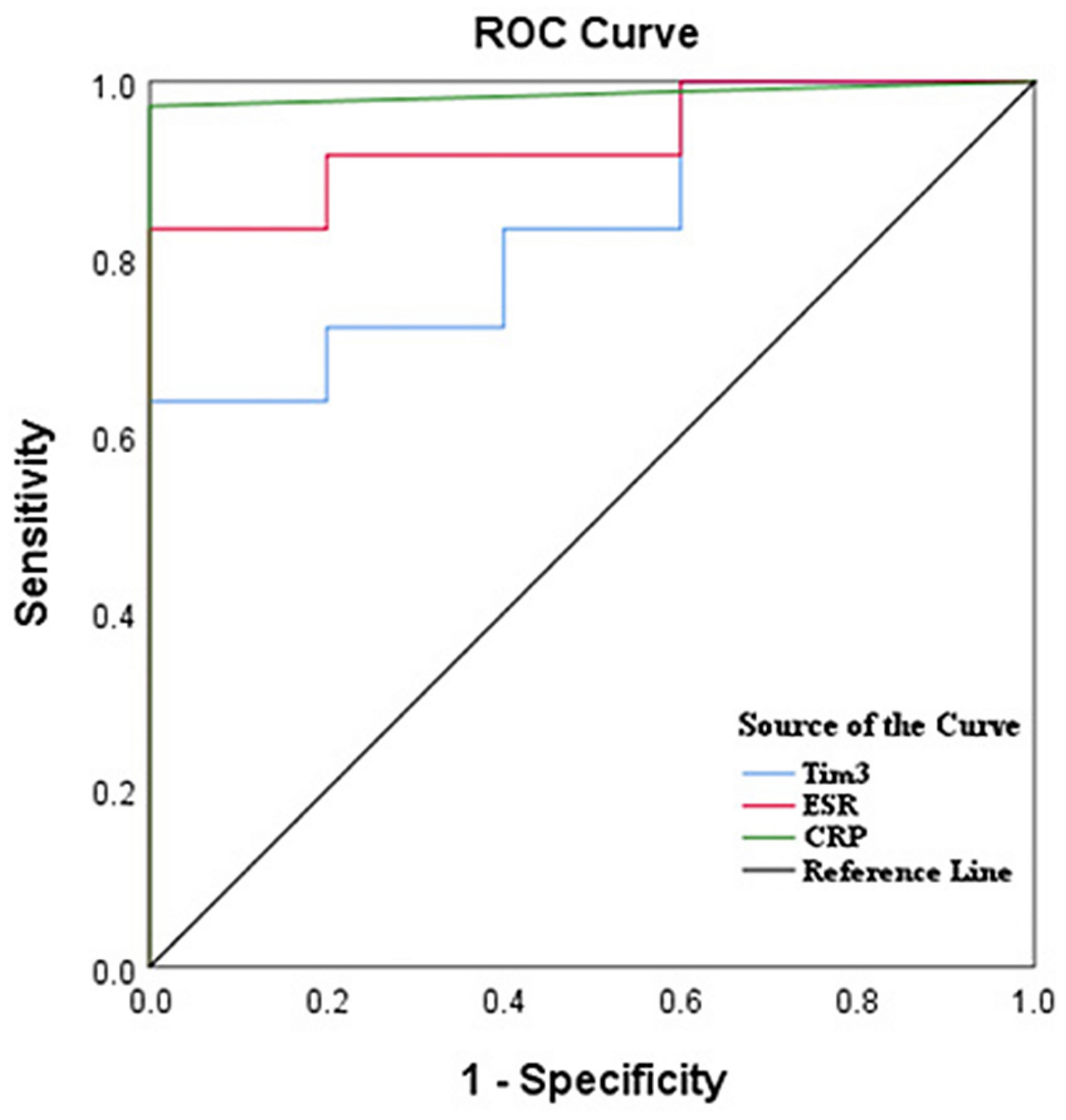
Tim-3 on neutrophils had a high diagnostic value for AS by ROC curve analysis. ROC curves of ESR, CRP, and Tim-3 for differentiating AS patients with active disease from inactive patients.

Receiver operating characteristic (ROC) analysis was utilized to assess the diagnostic value of severity in active AS patients compared to inactive patients. The results demonstrated that the area under the curve (AUC) for Tim-3, ESR, and CRP were 0.839 [95% confidence interval (CI): 0.686, 0.992], 0.933 (95% CI: 0.851, 1.000), and 0.986 (95% CI: 0.953, 1.00), respectively. Although the AUC for Tim-3 was slightly lower than that of ESR and CRP, it still exhibited diagnostic value for assessing AS severity. Additionally, the optimal cutoff value for Tim-3 in predicting active patients was determined to be 313.5, with a specificity of 100% and a sensitivity of 69.7% (Figure 4; Table 2).

**Table 2:** ROC curves of Tim3, ESR, and CRP for differentiating active AS from inactive patients.

**Test result variable(s)**	**AUC**	**95% CI**	**Optimal cutoff value**	**Sensitivity**	**Specificity**
Tim 3	0.839	0.686–0.992	313.5	0.697	1.000
ESR	0.933	0.851–1.000	14.5	0.758	1.000
CRP	0.986	0.953–1.000	2.35	0.939	0.875

### 3.6 The level of Tim-3 MFI of neutrophils was decreased after treatment

**Figure 5:**
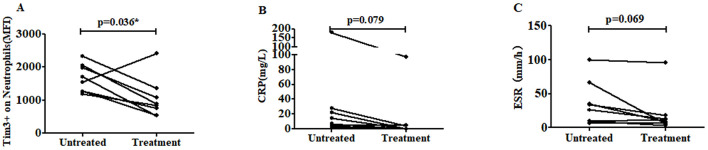
The level of Tim-3 MFI on neutrophils was decreased after treatment. **(A)** The level of Tim-3 MFI on neutrophils was shown in eight AS patients following regular treatment with NSAIDs, bDMARDs and csDMARDs (*p* = 0.036). **(B)** The level of CRP was decreased after treatment. The CRP levels in eight AS patients were measured before and after regular treatment with NSAIDs, bDMARDs, and csDMARDs (*p* = 0.079). **(C)** The level of ESR was decreased after treatment. The ESR levels in eight AS patients were measured before and after regular treatment with NSAIDs, bDMARDs, and csDMARDs (*p* = 0.069). ^*^P < 0.05.

Subsequently, we conducted a 1-month follow-up evaluation in eight patients receiving regular treatment with non-steroidal anti-inflammatory drugs (NSAIDs), biological disease-modifying antirheumatic drugs (bDMARDs), and conventional synthetic DMARDs (csDMARDs). The clinical response and Tim-3 MFI of neutrophils were monitored throughout the treatment period. Remarkably, the Tim-3 MFI of neutrophils decreased post-treatment in seven out of the eight AS patients, compared to the levels observed before treatment. Only one patient showed no significant change (Figure 5A). Furthermore, we evaluated the levels of inflammatory markers, including CRP and ESR, before and after treatment. Although the reduction of CRP did not reach statistical significance (*p* = 0.079), a consistent downward trend was observed in all eight patients (Figure 5B). Similarly, ESR levels showed a decline following treatment, although the difference was not statistically significant (*p* = 0.069) (Figure 5C). These findings suggest that Tim-3 MFI on neutrophils, along with inflammatory markers such as CRP and ESR, tends to decrease after treatment. This indicates a potential association between Tim-3 expression and disease activity in AS patients, further supporting its role as a biomarker for monitoring therapeutic response.

### 3.7 The MFI of Tim-3 of neutrophils was not associated with alterations in MRI

In recent decades, MRI has emerged as a crucial and sensitive tool for detecting early lesions in the sacroiliac joint, axial joint, and peripheral arthritis in AS (24). Early diagnosis and treatment based on MRI findings can lead to improved prognosis and reversible disease changes. For quantitative assessment of inflammation in the hip and sacroiliac joint, we utilized the HIMRISS and the SPARCC MRI index.

**Figure 6:**
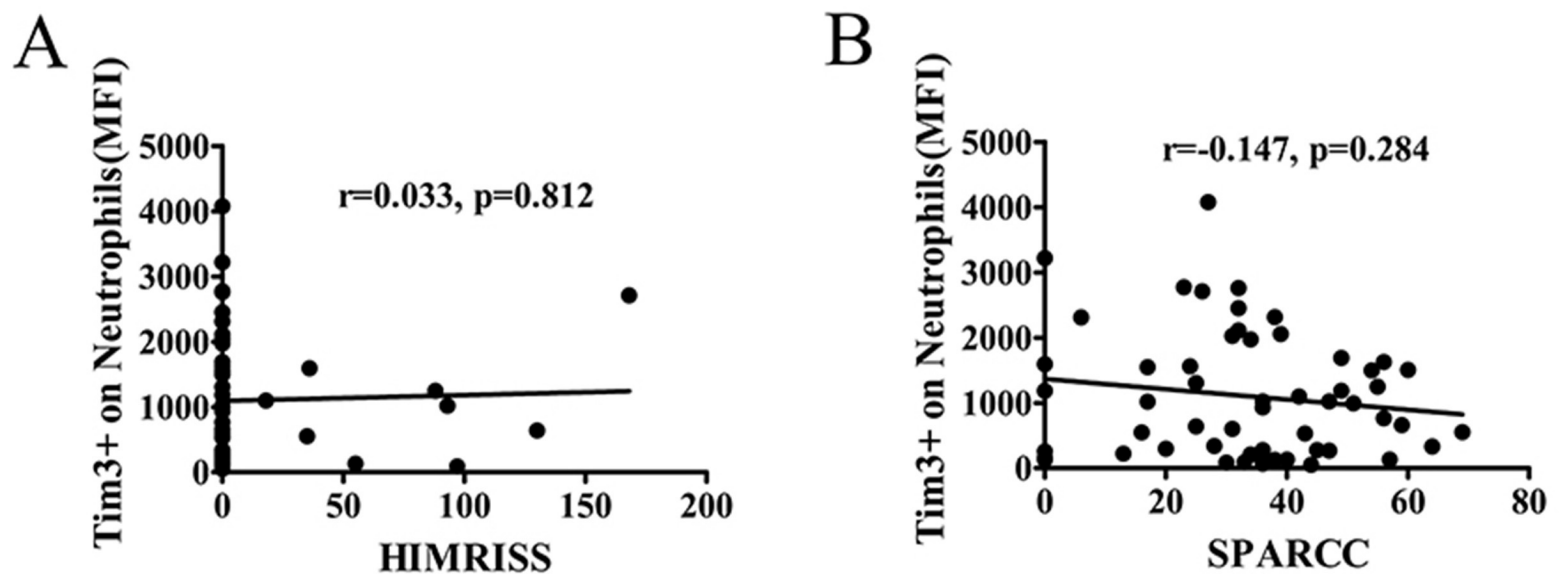
The MFI of Tim-3 on neutrophils was not associated with alterations in MRI. The correlation coefficient between the level of Tim-3 on neutrophils and MRI score were analyzed. The inflammation of the hip in MRI was assessed using the hip inflammation MRI scoring system (HIMRISS). The inflammation of the SJ in MRI was assessed by the Spondyloarthritis Research Consortium of Canada (SPARCC) MRI index. **(A)** The MFI of Tim-3 on neutrophils in AS patients was not associated with HIMRISS. **(B)** The MFI of Tim-3 on neutrophils in AS patients was not associated with SPARCC.

To explore the potential correlation between lesions observed in MRI of the hip and sacroiliac joint and the Tim-3 MFI of neutrophils, we conducted an analysis of the relationship between the Tim-3 MFI of neutrophils and HIMRISS or SPARCC. However, our findings revealed no significant correlation between the level of Tim-3 MFI of neutrophils and either the HIMRISS or SPARCC MRI index for the sacroiliac joint (Figures 6A, B).

## 4 Discussion

In recent years, there has been a growing recognition of the role played by the innate immune system in autoimmune diseases. Neutrophils are widely acknowledged as one of the primary effector cells of the human innate immune system, constituting the most abundant leukocyte population. Recent studies have shown an increasing interest in characterizing neutrophils and their involvement in the interaction and regulation of the adaptive immune response (25, 26). Emerging evidence over the last few decades has emphasized the critical role of neutrophils in the progression of AS (27). However, our understanding of the immunomodulatory roles and mechanisms of neutrophils in the initiation and progression of AS remains limited.

It is well-established that the expression of costimulatory molecules plays a pivotal role in determining the activation status and function of immune cells. Certain costimulatory molecules, particularly immunosuppressive ones like PD-1, programmed death-ligand 1 (PD-L1), and Tim-3, have been reported to exhibit aberrant expression in peripheral T cells, B cells, monocytes, or natural killer cells in AS patients. In this study, we conducted the first investigation into the expression of PD-1 and Tim-3 on neutrophils from AS patients, revealing a notable increase in the MFI of Tim-3 on neutrophils in AS patients compared to HC. Furthermore, our findings indicated that the Tim-3 expression level, as measured by the MFI of Tim-3 staining on neutrophils, was correlated with disease activity and severity of AS. Previous studies have reported no expression of Tim-3 on neutrophils by comparing the percentage of Tim-3 on neutrophils between lupus patients and healthy volunteers (25). Similarly, our study found no difference in the percentage of Tim-3-expressing neutrophils between AS patients and HC. However, the comparison of the Tim-3 MFI of neutrophils in the two groups suggested an increased Tim-3 MFI of neutrophils in AS patients. Previous research has also indicated that inflammatory stimuli can increase the Tim-3 MFI of neutrophils (28). Since Tim-3 exhibits weak fluorescence intensity, weak positive data would be lost if percentage statistics were used. Moreover, both the percentage and MFI of PD-1 on neutrophils were not significantly increased in AS patients compared with healthy individuals.

In this study, we observed a positive association between the level of Tim-3 MFI of neutrophils and ESR and CRP, suggesting a potential correlation between Tim-3 MFI of neutrophils and disease activity in AS. Subsequent analysis based on ASDAS classification of AS patients further supported this notion, revealing a positive correlation between Tim-3 MFI of neutrophils and ASDAS. Thus, we established a link between Tim-3 MFI of neutrophils and disease activity in AS. However, no correlation was observed between Tim-3 MFI of neutrophils and BASDAI or Bath Ankylosing Spondylitis Functional Index (BASFI). This discrepancy may be attributed to the limited sensitivity of BASDAI and BASFI in capturing systemic inflammation. Consequently, we utilized the more sensitive ASDAS index to distinguish Tim-3 expression in active and inactive AS patients. Further analysis revealed that Tim-3 expression was also not associated with imaging data, suggesting that Tim-3 may not directly contribute to disease activity, dysfunction, or joint structural changes unique to ankylosing spondylitis. BASDAI primarily assesses patients' subjective symptoms, such as fatigue and pain, while BASFI focuses on functional limitations. Imaging data, on the other hand, directly reflect morphological changes in the joints. The lack of association between Tim-3 and these indicators implies that Tim-3 may not be a direct driver of disease activity and functional impairment in ankylosing spondylitis. Instead, its role may be more related to systemic inflammation rather than specifically targeting the joint lesions that are characteristic of ankylosing spondylitis. Our findings demonstrated that the MFI of Tim-3 of neutrophils from patients with active AS was significantly higher compared to inactive AS patients. ROC curve analysis indicated that Tim-3 on neutrophils had a high diagnostic value for assessing the severity of AS. Taken together, these results suggest a correlation between Tim-3 MFI of neutrophils and disease severity in AS.

To further explore the clinical relevance of Tim-3 levels in neutrophils in AS, we conducted a 1-month follow-up assessment of eight AS patients undergoing regular treatment with non-steroidal anti-inflammatory drugs, bDMARDs, and csDMARDs. The data revealed a decrease in the MFI of Tim-3 in neutrophils in seven out of these eight patients following the 30-day treatment period, with only one patient showing no significant change. These findings suggest that Tim-3 levels may serve as a potential indicator for evaluating therapeutic efficacy. The data showed that patients undergoing anti-inflammatory and disease-modifying therapies exhibited decreased ESR and CRP (Figure 5). The patient who did not show a reduction in Tim-3 expression, there was still a corresponding decrease in CRP and ESR. Neutrophils are an important part of the human immune system and undergo changes in the early stages of many diseases. Assessing the level of Tim-3 on neutrophils may help detect abnormalities in the early stages of the disease, even before symptoms become obvious. Taking infectious diseases and autoimmune diseases as examples, although both may have inflammatory manifestations, in infectious diseases, the expression of Tim-3 on neutrophils may change earlier than traditional inflammatory indicators such as CRP, providing a more sensitive indicator for early diagnosis and also helping doctors make differential diagnoses to avoid misdiagnosis. During the disease remission period, monitoring the level of Tim-3 on neutrophils can help predict whether the disease is likely to relapse. If the level of Tim-3 abnormally increases, it may indicate that the immune system is reactivated and the disease has a risk of recurrence, allowing for the adoption of preventive measures in advance. Tim-3 is an important immune regulatory molecule. In immunotherapy, regular assessment of the level of Tim-3 on neutrophils can promptly determine whether the treatment is effective. If the treatment is effective, the level of Tim-3 may gradually return to normal or approach the normal range. Conversely, if the level of Tim-3 remains abnormal, it may be necessary to consider changing the treatment plan.

The results indicated that the expression of Tim-3 was not correlated with the cell count in routine blood tests, suggesting that changes in Tim-3 levels in neutrophils were not influenced by peripheral blood cell counts, including leukocytes or neutrophils. Furthermore, we extended our analysis to other hematological parameters, particularly in the context of anemia, which is a common comorbidity in AS. AS is considered one of the types of axial spondyloarthritis. According to research statistics, the incidence of anemia in axial spondyloarthritis ranges from 5.6 to 47.9%. In our study, the incidence of anemia in patients with ankylosing spondylitis was 27.4%, which is consistent with the incidence reported in the literature. To explore whether anemia might influence our findings, we analyzed the correlation between red blood cell (RBC) count, hemoglobin (HGB) levels, and the MFI of Tim-3 on neutrophils. The results showed that Tim-3 expression was not correlated with either RBC count or HGB levels (Supplementary Figure 1), leading us to conclude that there is no evidence suggesting anemia affected the results. Additionally, no correlation was found between Tim-3 expression and changes in imaging. This lack of association may be attributed to the fact that Tim-3 levels in neutrophils reflect acute-phase changes, while imaging changes in AS primarily represent chronic alterations.

Previously, immunosuppressive subsets of neutrophils have been identified in human peripheral blood in autoimmune diseases and cancer (29), as well as the release of enzymatic or chemical mediators such as arginase-1 or ROS (29, 30). This is speculated to function as a negative feedback mechanism, preventing potential tissue damage caused by an excessive immune response. Given that the immunosuppressive characteristics of Tim-3 and its expression on neutrophils are related to the disease activity and severity of AS, our study suggests that the upregulation of Tim-3 expression on neutrophils might serve as a negative feedback mechanism aimed at averting potential tissue damage resulting from exaggerated autoimmune responses in AS patients.

## 5 Conclusion

Our study provides the first evidence of elevated Tim-3 expression on neutrophils in AS patients, highlighting its potential role as a biomarker of systemic inflammation and disease activity. The correlation between Tim-3 and inflammatory markers (CRP and ESR) suggests that Tim-3 may contribute to the immune dysregulation observed in AS. However, its lack of association with functional impairment and structural damage indicates that it primarily reflects systemic inflammation rather than AS-specific pathology. The potential clinical applications of Tim-3 monitoring include early disease detection, relapse prediction, and treatment response assessment. Further research is needed to elucidate the mechanistic role of Tim-3 in neutrophil-mediated immune regulation and its potential as a therapeutic target in AS.

## Data Availability

The raw data supporting the conclusions of this article will be made available by the authors, without undue reservation.

## Supplementary material

Supplementary Figure 1 Correlation between Tim-3 expression on neutrophils and hematological parameters in AS patients. **(A)** Correlation between Tim-3 MFI on neutrophils and lymphocyte (LY) count (*r* = 0.217, *p* = 0.080). **(B)** Correlation between Tim-3 MFI on neutrophils and monocyte (MONO) count (*r* = 0.026, *p* = 0.840). **(C)** Correlation between Tim-3 MFI on neutrophils and platelet (PLT) count. (*r* = 0.059, *p* = 0.638). **(D)** Correlation between Tim-3 MFI on neutrophils and red blood cell (RBC) count (*r* = −0.125, *p* = 0.321). **(E)** Correlation between Tim-3 MFI on neutrophils and hemoglobin (HGB) levels. (*r* = −0.080, *p* = 0.529). **(F)** Correlation between Tim-3 MFI on neutrophils and Hematocrit (HCT) levels. (*r* = −0.076, *p* = 0.550). Data are presented as scatter plots with Pearson correlation coefficients (*r*) and *p*-values.
